# 5-Cyclo­pentyl-2-(3-fluoro­phen­yl)-3-methyl­sulfinyl-1-benzofuran

**DOI:** 10.1107/S1600536812025482

**Published:** 2012-06-13

**Authors:** Hong Dae Choi, Pil Ja Seo, Uk Lee

**Affiliations:** a Department of Chemistry, Dongeui University, San 24 Kaya-dong, Busanjin-gu, Busan 614-714, Republic of Korea; b Department of Chemistry, Pukyong National University, 599-1 Daeyeon 3-dong, Nam-gu, Busan 608-737, Republic of Korea

## Abstract

In the title compound, C_20_H_19_FO_2_S, the benzofuran fragment is essentially planar, with a largest deviation from the mean plane of 0.026 (2) Å. The benzene ring makes a dihedral angle of 30.72 (12)° with this plane. The cyclo­pentyl group adopts an envelope conformation, with the α-C atom as the flap. This atom is disordered over two sites with occupancy factors of 0.803 (16) and 0.197 (16). In the crystal, mol­ecules are linked by weak C—H⋯O, C—H⋯π and C—F⋯π [3.257 (3) Å] inter­actions.

## Related literature

For the crystal structures of related compounds, see: Choi *et al.* (2011 ▶); Seo *et al.* (2011 ▶).
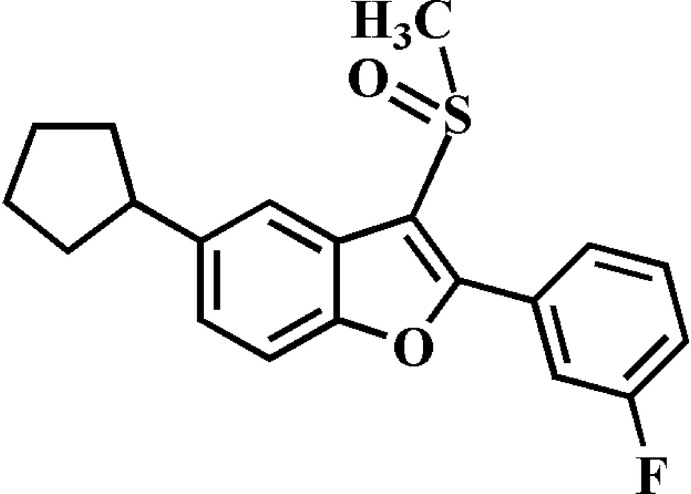


## Experimental

### 

#### Crystal data

C_20_H_19_FO_2_S
*M* *_r_* = 342.41
Monoclinic, 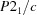

*a* = 6.1024 (3) Å
*b* = 25.3030 (11) Å
*c* = 10.6840 (5) Å
β = 90.231 (1)°
*V* = 1649.69 (13) Å^3^
*Z* = 4
Mo *K*α radiation
μ = 0.22 mm^−1^
*T* = 173 K
0.40 × 0.25 × 0.22 mm

#### Data collection

Bruker SMART APEXII CCD diffractometer
Absorption correction: multi-scan (*SADABS*; Bruker, 2009 ▶) *T*_min_ = 0.919, *T*_max_ = 0.954
13224 measured reflections
2905 independent reflections
2568 reflections with *I* > 2σ(*I*)
*R*_int_ = 0.027

#### Refinement

*R*[*F* ^2^ > 2σ(*F* ^2^)] = 0.060
*wR*(*F* ^2^) = 0.165
*S* = 1.02
2905 reflections
228 parameters
30 restraints
H-atom parameters constrained
Δρ_max_ = 1.35 e Å^−3^
Δρ_min_ = −0.55 e Å^−3^

### 

Data collection: *APEX2* (Bruker, 2009 ▶); cell refinement: *SAINT* (Bruker, 2009 ▶); data reduction: *SAINT*; program(s) used to solve structure: *SHELXS97* (Sheldrick, 2008 ▶); program(s) used to refine structure: *SHELXL97* (Sheldrick, 2008 ▶); molecular graphics: *ORTEP-3* (Farrugia, 1997 ▶) and *DIAMOND* (Brandenburg, 1998 ▶); software used to prepare material for publication: *SHELXL97*.

## Supplementary Material

Crystal structure: contains datablock(s) global, I. DOI: 10.1107/S1600536812025482/yk2058sup1.cif

Structure factors: contains datablock(s) I. DOI: 10.1107/S1600536812025482/yk2058Isup2.hkl

Supplementary material file. DOI: 10.1107/S1600536812025482/yk2058Isup3.cml

Additional supplementary materials:  crystallographic information; 3D view; checkCIF report

## Figures and Tables

**Table 1:** Hydrogen-bond geometry (Å, °) *Cg*1 and *Cg*2 are the centroids of the C1–C3/C8/O1 furan ring and the C2–C7 benzene ring, respectively.

*D*—H⋯*A*	*D*—H	H⋯*A*	*D*⋯*A*	*D*—H⋯*A*
C19—H19⋯O2^i^	0.95	2.52	3.326 (4)	143
C20—H20*B*⋯O2^i^	0.98	2.47	3.279 (4)	140
C9—H9*A*⋯*Cg*1^ii^	1.00	2.76	3.626 (4)	145
C15—H15⋯*Cg*2^iii^	0.95	2.94	3.461 (4)	116

Symmetry codes: (i) 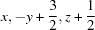; (ii) 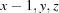; (iii) 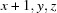.

**Fig. 1.**
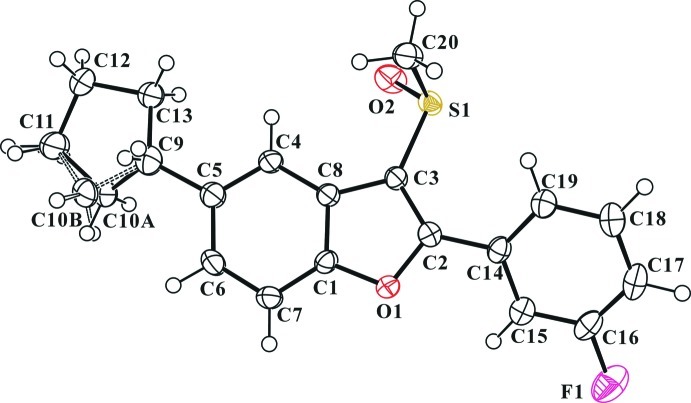
The molecular structure of the title compound with the atom numbering scheme. Displacement ellipsoids are drawn at the 50% probability level. The occupancy factors of C10A and C10B atoms are 0.803 (16) and 0.197 (16), respectively.

**Fig. 2.**
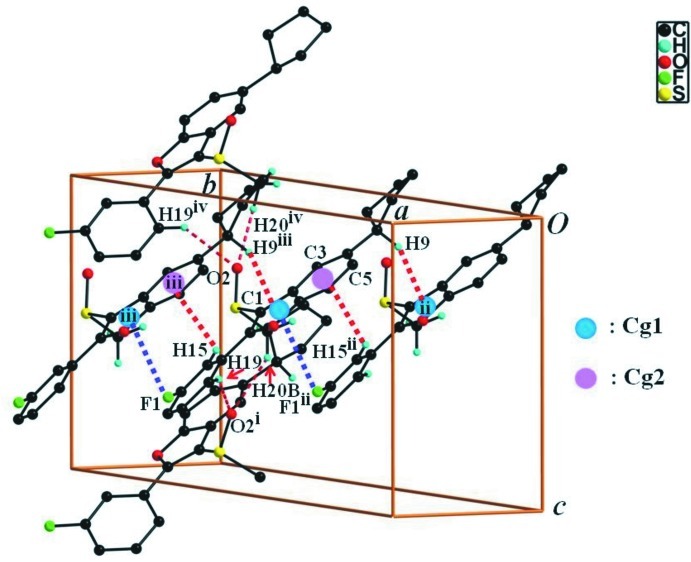
A view of the C—H···O, C—H···π and C—F···π interactions (dotted lines) in the crystal structure of the title compound. H atoms nonparticipating in hydrogen bonding are omitted for clarity. [Symmetry codes: (i) x, - y + 3/2, z + 1/2; (ii) x - 1, y, z; (iii) x + 1, y, z; (iv) x, - y + 3/2, z - 1/2.]

## supplementary crystallographic information

### Comment

As a part of our ongoing study of 5-cyclopentyl-3-methylsulfinyl-1-benzofuran derivatives containing 2-phenyl (Choi *et al.*, 2011) and 2-(4-fluorophenyl) (Seo *et al.*, 2011) substituents, we report herein the crystal structure of the title compound.

In the title molecule (Fig. 1), the benzofuran unit is essentially planar, with a mean deviation of 0.017 (2) Å from the least-squares plane defined by the nine constituent atoms. The cyclopentyl ring has an envelope conformation with the C10 atom as a flop. This atom is disordered over two sites, C10A and C10B, with occupancy factors of 0.803 (16) and 0.197 (16), respectively. The dihedral angle between the 3-fluorophenyl group and the mean plane of the benzofuran fragment is 30.7 (1)°. In the crystal structure, molecules are connected by weak C—H···O and C—H···π interactions (Table 1, Cg1 and Cg2 are the centroids of the C1–C3/C8/O1 furan ring and the C2–C7 benzene ring, respectively). The crystal packing (Fig. 2) also exhibits C—F···π interactions between the fluorine atom and the furan ring of an adjacent molecule, with a C16—F1···Cg1^iii^ distance of 3.257 (3) Å.

### Experimental

3-Chloroperoxybenzoic acid (77%, 224 mg, 1.0 mmol) was added in small portions to a stirred solution of 5-cyclopentyl-2-(3-fluorophenyl)-3-methylsulfanyl-1-benzofuran (293 mg, 0.9 mmol) in dichloromethane (30 mL) at 273 K. After being stirred at room temperature for 5h, the mixture was washed with saturated sodium hydrocarbonate solution and the organic layer was separated, dried over magnesium sulfate, filtered and concentrated at reduced pressure. The residue was purified by column chromatography (hexane-ethyl acetate, 1:2 v/v) to afford the title compound as a colorless solid [yield 81%, m.p. 430-431 K; *R*_f_ = 0.56 (hexane-ethyl acetate, 1:2 v/v)]. Single crystals suitable for X-ray diffraction were prepared by slow evaporation of a solution of the title compound in ethyl acetate at room temperature.

### Refinement

All H atoms were positioned geometrically and refined using a riding model, with C—H = 0.95 Å for the aryl, 1.00 Å for the methine, 0.99 Å for the methylene, and 0.98 Å for the methyl H atoms. U_iso_(H) = 1.2*U*_eq_(C) for the aryl, methine, and methylene H atoms, and 1.5*U*_eq_(C) for the methyl H atoms. The methyl group was allowed to rotate during the refinement.

The C10 atom of the cyclopentyl ring is disordered over two positions. The site occupancy factors were refined to 0.807 (16) (part A) and 0.193 (16) (part B). The distances of equivalent C-C pairs were restrained to 1.525 (4) Å and 0.001 Å using command DFIX and SADI, respectively, and displacement ellipsoids of C10A and C10B were restrained using command ISOR and DELU.

### Crystal data

**Table:**

C_20_H_19_FO_2_S	*F*(000) = 720
*M**_r_* = 342.41	*D*_x_ = 1.379 Mg m^−^^3^
Monoclinic, *P*2_1_/*c*	Mo *K*α radiation, λ = 0.71073 Å
Hall symbol: -P 2ybc	Cell parameters from 5280 reflections
*a* = 6.1024 (3) Å	θ = 2.5–28.2°
*b* = 25.3030 (11) Å	µ = 0.22 mm^−^^1^
*c* = 10.6840 (5) Å	*T* = 173 K
β = 90.231 (1)°	Block, colourless
*V* = 1649.69 (13) Å^3^	0.40 × 0.25 × 0.22 mm
*Z* = 4	

### Data collection

**Table:**

Bruker SMART APEXII CCD diffractometer	2905 independent reflections
Radiation source: rotating anode	2568 reflections with *I* > 2σ(*I*)
Graphite multilayer monochromator	*R*_int_ = 0.027
Detector resolution: 10.0 pixels mm^-1^	θ_max_ = 25.0°, θ_min_ = 2.1°
φ and ω scans	*h* = −7→7
Absorption correction: multi-scan (*SADABS*; Bruker, 2009)	*k* = −30→30
*T*_min_ = 0.919, *T*_max_ = 0.954	*l* = −12→12
13224 measured reflections	

### Refinement

**Table:**

Refinement on *F*^2^	Primary atom site location: structure-invariant direct methods
Least-squares matrix: full	Secondary atom site location: difference Fourier map
*R*[*F*^2^ > 2σ(*F*^2^)] = 0.060	Hydrogen site location: difference Fourier map
*wR*(*F*^2^) = 0.165	H-atom parameters constrained
*S* = 1.02	*w* = 1/[σ^2^(*F*_o_^2^) + (0.087*P*)^2^ + 2.8614*P*] where *P* = (*F*_o_^2^ + 2*F*_c_^2^)/3
2905 reflections	(Δ/σ)_max_ < 0.001
228 parameters	Δρ_max_ = 1.35 e Å^−^^3^
30 restraints	Δρ_min_ = −0.55 e Å^−^^3^

### Special details

**Table:**

Geometry. All s.u.'s (except the s.u. in the dihedral angle between two l.s. planes) are estimated using the full covariance matrix. The cell s.u.'s are taken into account individually in the estimation of s.u.'s in distances, angles and torsion angles; correlations between s.u.'s in cell parameters are only used when they are defined by crystal symmetry. An approximate (isotropic) treatment of cell s.u.'s is used for estimating s.u.'s involving l.s. planes.
Refinement. Refinement of F^2^ against ALL reflections. The weighted R-factor wR and goodness of fit S are based on F^2^, conventional R-factors R are based on F, with F set to zero for negative F^2^. The threshold expression of F^2^ > 2sigma(F^2^) is used only for calculating R-factors(gt) etc. and is not relevant to the choice of reflections for refinement. R-factors based on F^2^ are statistically about twice as large as those based on F, and R- factors based on ALL data will be even larger.

### Fractional atomic coordinates and isotropic or equivalent isotropic displacement parameters (Å^2^)

**Table:**

	*x*	*y*	*z*	*U*_iso_*/*U*_eq_	Occ. (<1)
S1	0.49517 (11)	0.72428 (3)	0.41704 (6)	0.0274 (2)	
O1	0.5749 (3)	0.56958 (7)	0.45210 (17)	0.0261 (4)	
O2	0.4494 (4)	0.74049 (8)	0.28532 (19)	0.0403 (6)	
F1	1.2882 (3)	0.56555 (10)	0.6805 (2)	0.0617 (6)	
C1	0.3984 (4)	0.57030 (11)	0.3714 (2)	0.0245 (6)	
C2	0.6189 (4)	0.62149 (11)	0.4815 (2)	0.0242 (6)	
C3	0.4786 (4)	0.65466 (11)	0.4202 (2)	0.0234 (6)	
C4	0.1509 (5)	0.63050 (11)	0.2681 (3)	0.0289 (6)	
H4	0.1049	0.6654	0.2488	0.035*	
C5	0.0407 (5)	0.58719 (12)	0.2184 (3)	0.0344 (7)	
C6	0.1179 (5)	0.53590 (12)	0.2453 (3)	0.0356 (7)	
H6	0.0429	0.5066	0.2096	0.043*	
C7	0.2976 (5)	0.52630 (11)	0.3211 (3)	0.0306 (6)	
H7	0.3486	0.4915	0.3376	0.037*	
C8	0.3310 (4)	0.62182 (10)	0.3474 (2)	0.0233 (6)	
C9	−0.1701 (6)	0.59386 (12)	0.1427 (3)	0.0448 (8)	
H9A	−0.2944	0.5837	0.1984	0.054*	0.803 (16)
H9B	−0.2753	0.5944	0.2142	0.054*	0.197 (16)
C10A	−0.1826 (10)	0.5586 (2)	0.0271 (5)	0.0527 (19)	0.803 (16)
H10A	−0.2053	0.5212	0.0505	0.063*	0.803 (16)
H10B	−0.0476	0.5615	−0.0236	0.063*	0.803 (16)
C10B	−0.283 (3)	0.5505 (2)	0.0671 (11)	0.035 (3)	0.197 (16)
H10C	−0.3989	0.5330	0.1167	0.043*	0.197 (16)
H10D	−0.1763	0.5236	0.0390	0.043*	0.197 (16)
C11	−0.3803 (8)	0.58042 (15)	−0.0439 (5)	0.0776 (16)	
H11A	−0.3541	0.5794	−0.1352	0.093*	0.803 (16)
H11B	−0.5127	0.5593	−0.0252	0.093*	0.803 (16)
H11C	−0.2866	0.5788	−0.1192	0.093*	0.197 (16)
H11D	−0.5234	0.5637	−0.0628	0.093*	0.197 (16)
C12	−0.4100 (5)	0.63712 (13)	0.0002 (3)	0.0377 (7)	
H12A	−0.5526	0.6415	0.0428	0.045*	
H12B	−0.4034	0.6619	−0.0713	0.045*	
C13	−0.2206 (5)	0.64710 (12)	0.0912 (3)	0.0377 (7)	
H13A	−0.0923	0.6620	0.0472	0.045*	
H13B	−0.2649	0.6717	0.1586	0.045*	
C14	0.7931 (4)	0.62945 (11)	0.5734 (2)	0.0258 (6)	
C15	0.9653 (4)	0.59298 (12)	0.5813 (3)	0.0297 (6)	
H15	0.9718	0.5635	0.5263	0.036*	
C16	1.1247 (5)	0.60094 (13)	0.6706 (3)	0.0341 (7)	
C17	1.1235 (5)	0.64302 (14)	0.7524 (3)	0.0390 (8)	
H17	1.2369	0.6474	0.8126	0.047*	
C18	0.9526 (5)	0.67855 (13)	0.7442 (3)	0.0380 (7)	
H18	0.9485	0.7079	0.7995	0.046*	
C19	0.7870 (5)	0.67197 (12)	0.6566 (3)	0.0304 (6)	
H19	0.6690	0.6964	0.6530	0.036*	
C20	0.2521 (5)	0.73842 (12)	0.5038 (3)	0.0339 (7)	
H20A	0.2213	0.7764	0.4998	0.051*	
H20B	0.2733	0.7279	0.5913	0.051*	
H20C	0.1286	0.7188	0.4679	0.051*	

### Atomic displacement parameters (Å^2^)

**Table:**

	*U*^11^	*U*^22^	*U*^33^	*U*^12^	*U*^13^	*U*^23^
S1	0.0324 (4)	0.0216 (4)	0.0281 (4)	−0.0040 (3)	−0.0031 (3)	0.0021 (2)
O1	0.0283 (10)	0.0232 (9)	0.0266 (10)	0.0024 (7)	−0.0057 (8)	0.0012 (7)
O2	0.0606 (15)	0.0312 (11)	0.0290 (11)	−0.0028 (10)	0.0000 (10)	0.0099 (9)
F1	0.0414 (12)	0.0772 (16)	0.0665 (15)	0.0171 (11)	−0.0087 (10)	0.0048 (12)
C1	0.0263 (13)	0.0261 (14)	0.0210 (13)	0.0024 (11)	−0.0039 (10)	0.0022 (10)
C2	0.0254 (13)	0.0252 (13)	0.0219 (13)	−0.0018 (10)	0.0016 (10)	−0.0007 (10)
C3	0.0270 (13)	0.0226 (13)	0.0205 (13)	−0.0010 (10)	−0.0019 (10)	0.0010 (10)
C4	0.0356 (15)	0.0229 (14)	0.0280 (14)	0.0018 (11)	−0.0092 (12)	0.0030 (11)
C5	0.0420 (17)	0.0282 (15)	0.0330 (16)	−0.0005 (13)	−0.0125 (13)	0.0015 (12)
C6	0.0451 (18)	0.0238 (14)	0.0379 (17)	−0.0038 (12)	−0.0144 (14)	−0.0035 (12)
C7	0.0387 (16)	0.0218 (14)	0.0313 (15)	0.0026 (12)	−0.0070 (12)	0.0010 (11)
C8	0.0263 (13)	0.0225 (13)	0.0210 (13)	−0.0002 (10)	−0.0008 (10)	0.0008 (10)
C9	0.0461 (19)	0.0373 (18)	0.0509 (18)	−0.0018 (15)	−0.0180 (15)	0.0010 (14)
C10A	0.050 (3)	0.033 (2)	0.075 (3)	0.006 (2)	−0.039 (3)	−0.014 (2)
C10B	0.022 (7)	0.034 (6)	0.050 (6)	−0.006 (5)	0.009 (5)	−0.004 (4)
C11	0.084 (3)	0.041 (2)	0.107 (3)	0.016 (2)	−0.072 (3)	−0.018 (2)
C12	0.0324 (16)	0.0392 (17)	0.0416 (17)	0.0035 (13)	−0.0108 (13)	−0.0001 (14)
C13	0.0411 (17)	0.0322 (16)	0.0398 (17)	0.0042 (13)	−0.0130 (14)	−0.0041 (13)
C14	0.0231 (13)	0.0308 (14)	0.0236 (13)	−0.0023 (11)	−0.0011 (10)	0.0047 (11)
C15	0.0269 (14)	0.0348 (16)	0.0276 (14)	−0.0008 (12)	−0.0003 (11)	0.0019 (12)
C16	0.0223 (14)	0.0446 (18)	0.0354 (16)	0.0027 (12)	−0.0020 (12)	0.0099 (13)
C17	0.0310 (16)	0.055 (2)	0.0313 (16)	−0.0076 (14)	−0.0098 (12)	0.0031 (14)
C18	0.0391 (17)	0.0437 (18)	0.0311 (16)	−0.0048 (14)	−0.0067 (13)	−0.0056 (13)
C19	0.0298 (15)	0.0341 (15)	0.0274 (14)	−0.0005 (12)	−0.0022 (11)	−0.0008 (12)
C20	0.0394 (17)	0.0291 (15)	0.0332 (16)	0.0042 (13)	0.0000 (13)	−0.0008 (12)

### Geometric parameters (Å, º)

**Table:**

S1—O2	1.491 (2)	C10B—C11	1.5254 (17)
S1—C3	1.765 (3)	C10B—H10C	0.9900
S1—C20	1.789 (3)	C10B—H10D	0.9900
O1—C2	1.377 (3)	C11—C12	1.521 (5)
O1—C1	1.377 (3)	C11—H11A	0.9900
F1—C16	1.345 (4)	C11—H11B	0.9900
C1—C7	1.380 (4)	C11—H11C	0.9900
C1—C8	1.390 (4)	C11—H11D	0.9900
C2—C3	1.364 (4)	C12—C13	1.528 (4)
C2—C14	1.458 (4)	C12—H12A	0.9900
C3—C8	1.449 (4)	C12—H12B	0.9900
C4—C5	1.390 (4)	C13—H13A	0.9900
C4—C8	1.402 (4)	C13—H13B	0.9900
C4—H4	0.9500	C14—C19	1.396 (4)
C5—C6	1.410 (4)	C14—C15	1.401 (4)
C5—C9	1.526 (4)	C15—C16	1.375 (4)
C6—C7	1.383 (4)	C15—H15	0.9500
C6—H6	0.9500	C16—C17	1.377 (5)
C7—H7	0.9500	C17—C18	1.379 (5)
C9—C13	1.487 (4)	C17—H17	0.9500
C9—C10A	1.5249 (16)	C18—C19	1.386 (4)
C9—C10B	1.5250 (17)	C18—H18	0.9500
C9—H9A	1.0000	C19—H19	0.9500
C9—H9B	1.0000	C20—H20A	0.9800
C10A—C11	1.5254 (17)	C20—H20B	0.9800
C10A—H10A	0.9900	C20—H20C	0.9800
C10A—H10B	0.9900		
			
O2—S1—C3	106.38 (12)	C12—C11—C10A	106.4 (3)
O2—S1—C20	106.37 (14)	C12—C11—H11A	110.5
C3—S1—C20	98.18 (13)	C10B—C11—H11A	133.6
C2—O1—C1	106.34 (19)	C10A—C11—H11A	110.5
O1—C1—C7	125.4 (2)	C12—C11—H11B	110.5
O1—C1—C8	111.0 (2)	C10B—C11—H11B	83.8
C7—C1—C8	123.6 (2)	C10A—C11—H11B	110.5
C3—C2—O1	110.9 (2)	H11A—C11—H11B	108.6
C3—C2—C14	133.8 (3)	C12—C11—H11C	111.1
O1—C2—C14	115.2 (2)	C10B—C11—H11C	112.7
C2—C3—C8	107.0 (2)	C10A—C11—H11C	86.0
C2—C3—S1	126.0 (2)	H11B—C11—H11C	128.0
C8—C3—S1	126.7 (2)	C12—C11—H11D	111.1
C5—C4—C8	119.0 (3)	C10B—C11—H11D	106.7
C5—C4—H4	120.5	C10A—C11—H11D	130.0
C8—C4—H4	120.5	H11A—C11—H11D	86.2
C4—C5—C6	119.2 (3)	H11C—C11—H11D	109.2
C4—C5—C9	121.4 (3)	C11—C12—C13	105.2 (2)
C6—C5—C9	119.3 (3)	C11—C12—H12A	110.7
C7—C6—C5	123.0 (3)	C13—C12—H12A	110.7
C7—C6—H6	118.5	C11—C12—H12B	110.7
C5—C6—H6	118.5	C13—C12—H12B	110.7
C1—C7—C6	116.0 (3)	H12A—C12—H12B	108.8
C1—C7—H7	122.0	C9—C13—C12	103.9 (2)
C6—C7—H7	122.0	C9—C13—H13A	111.0
C1—C8—C4	119.3 (2)	C12—C13—H13A	111.0
C1—C8—C3	104.8 (2)	C9—C13—H13B	111.0
C4—C8—C3	135.9 (2)	C12—C13—H13B	111.0
C13—C9—C5	118.0 (3)	H13A—C13—H13B	109.0
C13—C9—C10A	102.7 (3)	C19—C14—C15	119.4 (3)
C5—C9—C10A	113.8 (3)	C19—C14—C2	120.9 (2)
C13—C9—C10B	111.3 (3)	C15—C14—C2	119.6 (3)
C5—C9—C10B	125.4 (5)	C16—C15—C14	118.3 (3)
C13—C9—H9A	107.2	C16—C15—H15	120.9
C5—C9—H9A	107.2	C14—C15—H15	120.9
C10A—C9—H9A	107.2	F1—C16—C15	118.7 (3)
C10B—C9—H9A	77.8	F1—C16—C17	118.1 (3)
C13—C9—H9B	97.9	C15—C16—C17	123.2 (3)
C5—C9—H9B	98.0	C16—C17—C18	118.1 (3)
C10A—C9—H9B	126.7	C16—C17—H17	121.0
C10B—C9—H9B	97.2	C18—C17—H17	121.0
C9—C10A—C11	103.2 (3)	C17—C18—C19	120.9 (3)
C9—C10A—H10A	111.1	C17—C18—H18	119.5
C11—C10A—H10A	111.1	C19—C18—H18	119.5
C9—C10A—H10B	111.1	C18—C19—C14	120.1 (3)
C11—C10A—H10B	111.1	C18—C19—H19	119.9
H10A—C10A—H10B	109.1	C14—C19—H19	119.9
C9—C10B—C11	103.2 (3)	S1—C20—H20A	109.5
C9—C10B—H10C	111.1	S1—C20—H20B	109.5
C11—C10B—H10C	111.1	H20A—C20—H20B	109.5
C9—C10B—H10D	111.1	S1—C20—H20C	109.5
C11—C10B—H10D	111.1	H20A—C20—H20C	109.5
H10C—C10B—H10D	109.1	H20B—C20—H20C	109.5
C12—C11—C10B	105.9 (5)		
			
C2—O1—C1—C7	−178.4 (3)	C4—C5—C9—C10B	169.7 (9)
C2—O1—C1—C8	0.9 (3)	C6—C5—C9—C10B	−14.5 (10)
C1—O1—C2—C3	−1.1 (3)	C13—C9—C10A—C11	−40.4 (6)
C1—O1—C2—C14	176.1 (2)	C5—C9—C10A—C11	−169.1 (4)
O1—C2—C3—C8	0.9 (3)	C10B—C9—C10A—C11	70.6 (5)
C14—C2—C3—C8	−175.6 (3)	C13—C9—C10B—C11	7.3 (14)
O1—C2—C3—S1	−172.60 (18)	C5—C9—C10B—C11	−146.2 (7)
C14—C2—C3—S1	10.9 (4)	C10A—C9—C10B—C11	−70.6 (5)
O2—S1—C3—C2	137.4 (2)	C9—C10B—C11—C12	−24.6 (13)
C20—S1—C3—C2	−112.8 (3)	C9—C10B—C11—C10A	70.6 (5)
O2—S1—C3—C8	−34.9 (3)	C9—C10A—C11—C12	22.9 (7)
C20—S1—C3—C8	74.9 (3)	C9—C10A—C11—C10B	−70.6 (5)
C8—C4—C5—C6	−2.3 (4)	C10B—C11—C12—C13	33.3 (9)
C8—C4—C5—C9	173.6 (3)	C10A—C11—C12—C13	2.6 (6)
C4—C5—C6—C7	1.1 (5)	C5—C9—C13—C12	168.4 (3)
C9—C5—C6—C7	−174.9 (3)	C10A—C9—C13—C12	42.3 (4)
O1—C1—C7—C6	177.9 (3)	C10B—C9—C13—C12	12.7 (9)
C8—C1—C7—C6	−1.3 (4)	C11—C12—C13—C9	−27.9 (4)
C5—C6—C7—C1	0.7 (5)	C3—C2—C14—C19	28.7 (5)
O1—C1—C8—C4	−179.2 (2)	O1—C2—C14—C19	−147.7 (3)
C7—C1—C8—C4	0.1 (4)	C3—C2—C14—C15	−153.4 (3)
O1—C1—C8—C3	−0.3 (3)	O1—C2—C14—C15	30.2 (3)
C7—C1—C8—C3	179.0 (3)	C19—C14—C15—C16	−0.7 (4)
C5—C4—C8—C1	1.7 (4)	C2—C14—C15—C16	−178.7 (3)
C5—C4—C8—C3	−176.7 (3)	C14—C15—C16—F1	178.7 (3)
C2—C3—C8—C1	−0.4 (3)	C14—C15—C16—C17	−0.2 (4)
S1—C3—C8—C1	173.1 (2)	F1—C16—C17—C18	−178.4 (3)
C2—C3—C8—C4	178.2 (3)	C15—C16—C17—C18	0.5 (5)
S1—C3—C8—C4	−8.3 (5)	C16—C17—C18—C19	0.2 (5)
C4—C5—C9—C13	17.8 (5)	C17—C18—C19—C14	−1.1 (5)
C6—C5—C9—C13	−166.4 (3)	C15—C14—C19—C18	1.4 (4)
C4—C5—C9—C10A	138.2 (5)	C2—C14—C19—C18	179.2 (3)
C6—C5—C9—C10A	−45.9 (5)		

### Hydrogen-bond geometry (Å, º)

Cg1 and Cg2 are the centroids of the C1–C3/C8/O1 furan ring and the C2–C7 benzene ring, respectively.

**Table:**

*D*—H···*A*	*D*—H	H···*A*	*D*···*A*	*D*—H···*A*
C19—H19···O2^i^	0.95	2.52	3.326 (4)	143
C20—H20*B*···O2^i^	0.98	2.47	3.279 (4)	140
C9—H9*A*···*Cg*1^ii^	1.00	2.76	3.626 (4)	145
C15—H15···*Cg*2^iii^	0.95	2.94	3.461 (4)	116

Symmetry codes: (i) *x*, −*y*+3/2, *z*+1/2; (ii) *x*−1, *y*, *z*; (iii) *x*+1, *y*, *z*.
